# Comparison of the population excess fraction of *Chlamydia trachomatis* infection on pelvic inflammatory disease at 12-months in the presence and absence of chlamydia testing and treatment: Systematic review and retrospective cohort analysis

**DOI:** 10.1371/journal.pone.0171551

**Published:** 2017-02-15

**Authors:** Bethan Davies, Katy M. E. Turner, Stella Leung, B. Nancy Yu, Maria Frølund, Thomas Benfield, James Blanchard, Henrik Westh, on behalf of the Danish Chlamydia Study, Helen Ward

**Affiliations:** 1 Department of Infectious Disease Epidemiology, School of Public Health, Imperial College London, London, United Kingdom; 2 School of Veterinary Science, University of Bristol, Langford, Bristol, United Kingdom; 3 Department of Community Health Sciences, University of Manitoba, Winnipeg, Manitoba, Canada; 4 Epidemiology & Surveillance, Public Health Branch, Manitoba Health Healthy Living and Seniors, Winnipeg, Manitoba, Canada; 5 Department of Microbiology and Infection Control, Statens Serum Institut, Copenhagen, Denmark; 6 Department of Infectious Diseases, Hvidovre University Hospital, Hvidovre, Denmark; 7 Institute of Clinical Medicine, University of Copenhagen, Copenhagen, Denmark; 8 Department of Clinical Microbiology, Hvidovre University Hospital, Hvidovre, Denmark; University of California, San Francisco, Universit of California, Berkeley and the Childrens Hospital Oakland Research Institute, UNITED STATES

## Abstract

**Background:**

The impact of *Chlamydia trachomatis* (chlamydia) control on the incidence of pelvic inflammatory disease (PID) is theoretically limited by the proportion of PID caused by chlamydia. We estimate the population excess fraction (PEF) of treated chlamydia infection on PID at 12-months in settings with widespread chlamydia control (testing and treatment) and compare this to the estimated PEF of untreated chlamydia.

**Methods:**

We used two large retrospective population-based cohorts of women of reproductive age from settings with widespread chlamydia control to calculate the PEF of treated chlamydia on PID at 12-months. We undertook a systematic review to identify further studies that reported the risk of PID in women who were tested for chlamydia (infected and uninfected). We used the same method to calculate the PEF in eligible studies then compared all estimates of PEF.

**Results:**

The systematic review identified a single study, a randomised controlled trial of chlamydia screening (POPI-RCT). In the presence of testing and treatment <10% of PID at 12-months was attributable to treated (baseline) chlamydia infections (Manitoba: 8.86%(95%CI 7.15–10.75); Denmark: 3.84%(3.26–4.45); screened-arm POPI-RCT: 0.99%(0.00–29.06)). In the absence of active chlamydia treatment 26.44%(11.57–46.32) of PID at 12-months was attributable to untreated (baseline) chlamydia infections (deferred-arm POPI-RCT). The PEFs suggest that eradicating baseline chlamydia infections could prevent 484 cases of PID at 12-months per 100,000 women in the untreated setting and 13–184 cases of PID per 100,000 tested women in the presence of testing and treatment.

**Conclusion:**

Testing and treating chlamydia reduced the PEF of chlamydia on PID by 65% compared to the untreated setting. But in the presence of testing and treatment over 90% of PID could not be attributed to a baseline chlamydia infection. More information is needed about the aetiology of PID to develop effective strategies for improving the reproductive health of women.

## Introduction

Many high-income countries invest heavily in opportunistic testing or screening for *Chlamydia trachomatis* (chlamydia) with the aim of reducing the incidence of infection and post-infectious complications (including pelvic inflammatory disease (PID)).[1, 2] The strongest evidence for chlamydia testing comes from a meta-analysis of randomised-controlled trials (RCT) that demonstrates the benefit of testing young women (under 25 years). For an individual, the offer of a chlamydia test can reduce her risk of PID at 12-months by 32% (RR 0.68; 95%CI 0.49–0.94).[3] However, it has not yet been shown that widespread testing for chlamydia can impact on the incidence or prevalence of infection or complications in the population.

Opportunistic testing and screening are interventions applied at the level of the population. To estimate the potential population-level health impact requires information about the relative risk of PID in chlamydia-positive compared to chlamydia-negative women (“relative risk”) and the prevalence of chlamydia. The resulting parameter is the population excess fraction (PEF)—also known as the “population attributable risk” or “population aetiologic fraction”. It is an estimate of the proportion of PID that could be prevented in a population if a causal exposure (a positive chlamydia test) was eradicated (or more correctly, if the exposure-level in those categorised as exposed (chlamydia-positive) was reduced to the level in those categorised as non-exposed (chlamydia-negative)).

The PEF provides useful information for determining how to invest resources for maximal health gain. A recent modelling study, that combined data from multiple sources, estimates that in the absence of chlamydia control interventions 19.7% (95% CI 5.9–38.1) of PID is attributable to an untreated chlamydia infection.[4] The authors also estimate that the widely used policy of annual chlamydia testing in young adults can prevent a maximum of 61% of cases of chlamydia-associated PID.

We aim to estimate the PEF of diagnosed chlamydia on PID at 12-months in women from the general population who are tested for chlamydia (infected and uninfected) and followed up (for at least 12-months) for the outcome of PID. This will provide additional information from a real-world setting that can be used to inform modelling studies and investment in chlamydia control interventions.

## Methods

### Ethics statement

MWRSH cohort: Institutional approval for use of the administrative healthcare data was obtained from the Health Information Privacy Committee at Manitoba Health (study approval number: HIPC 2010/2011-48). Ethical approval for the use of pseudo-anonymized administrative health data was obtained from the Health Research Ethics Board at the University of Manitoba. All data were provided and hosted by the Manitoba Centre for Health Policy within the University of Manitoba. Denmark Chlamydia Study: The study was approved by the Danish Data Protection Agency (J.nr. 2010-41-4866 and J.nr. 2012-331-0228).

### Primary data analysis

We analysed two retrospective population-based cohorts with individual-level linked data on chlamydia and PID (Manitoba Women’s Reproductive and Sexual Health (MWRSH) cohort and Denmark Chlamydia Study) that have been described in full elsewhere.[5, 6]

Briefly, the MWRSH cohort is a birth cohort that links demographic information from the Province of Manitoba’s health insurance system (Manitoba Health Insurance Registry [7]), chlamydia test data from the Province’s main microbiology laboratory (Cadham Provincial Laboratory [8]) and healthcare presentations for PID (in-patient, out-patient and community setting) from the Province’s administrative healthcare datasets (Medical Claims and Hospital Separations Abstracts [9]) (Table 1). The dataset has complete ascertainment of chlamydia tests undertaken in public laboratories and diagnosed episodes of PID excluding those seen only at an Emergency Department. This cohort contains 147,258 female residents of Manitoba aged 12–24 years in 1992–1996 who were followed-up until 2008. In this analysis, the cohort was restricted to the 72,883 women who were tested for chlamydia and did not have a PID diagnosis before their first chlamydia test in the cohort.[6] All women entered the cohort on the date of their first chlamydia test during the study period. We defined chlamydia prevalence (*p*_*e*_) as the proportion of women who had a positive result at their first chlamydia test in the cohort. We classified women according to the result of this first test and determined the proportion of chlamydia-negative (*I*_*u*_) and chlamydia-positive (*I*_*e*_) women who had a healthcare presentation with PID by 12-months.

**Table 1:** Summary of the included studies.

Definitions	MWRSH Cohort, Manitoba Canada	Denmark Chlamydia Study	UK-based POPI-RCT
**Design**	Retrospective cohort of administrative data	Retrospective cohort of administrative data	Randomised controlled trial
**Population**	Manitoba residents	Denmark residents	Sexually active; non-pregnant; no chlamydia test <3/12; educational settings in London
**Age**	12–24 years in 1992–1996	15–44 years in 1995–2012	≥16-≤27 years at recruitment
**Entry to cohort**	First CT test from 1992 onwards at ≥12 years	First CT test from 1995 onwards at ≥15 years	Recruitment, 2004–2006
**Exit from cohort**	31/12/2008; 45^th^ birthday; leaving the province; death; diagnosis of PID	31/10/2012; 45^th^ birthday; leaving Denmark; death; diagnosis of PID	12 months
**Maximum follow-up**	16 years	17 years	1 year
**Size**	72,883	286,223	2,529
**CT test**	Any non-serological test performed ≥60 days after a previous test	Genital, rectal or urinary sample ≥30 days after previous test	Vaginal swab self-taken at recruitment
**CT positive**	Positive result at first test in cohort	Positive result at first test in cohort	Positive result on recruitment sample
**CT negative**	Negative result at first test in cohort	Negative result at first test in cohort and no positive tests during follow-up	Negative result on recruitment sample
**CT diagnostic test**	Chlamydiazyme test~ (1992–98); PACE 2 nucleic acid probe test^#^ or AMP-CT NAAT^+^ (1999–07); Aptima NAAT^ (2007–08)	Varied across public health laboratories in Denmark; Change from non-nucleic acid method to NAATs occurred in 1999/2000	Transcription Mediated Amplification*
**CT test ascertainment**	Complete from Cadham Provincial Laboratory	Complete from public health laboratories	Complete from baseline sample
**Definition of PID**	ICD-9 614–616.0; 016.6; 098.10; 098.15; 098.16; 098.17; 098.30; 098.35–7; 099.56; 098.86; ICD-10 N70-74.8; A56.1; A18.1; A51.4; A54.2; A52.7	ICD-10 A18.2; A51.4; A52.7; A54.2; A56.1; N70-74.8	Self-reported (or GP for non-respondents) symptoms of PID; treatment PID/UTI, laparoscopy or presentation with abdo/pelvic pain
**PID ascertainment**	Community, out-patient and in-patient	Out-patient, ED and in-patient	Not stated
**Population rate of gonorrhoea in women**	105 per 100,000 in 2013[10];	4.2 per 100,000 in 2011[11];	25.7 per 100,000 in 2012[12]

CT: chlamydia; NAAT: Nucleic Acid Amplification Test; PCR: Polymerase Chain Reaction; ED: Emergency Department

~Abbott Laboratory, Chicago IL

^#^GenProbe, San Diego CA for urethral /cervical

^+^GenProbe, San Diego CA for urine

^GenProbe, San Diego CA

*TMA; Gen-Probe, San Diego, CA.

The Denmark Chlamydia Study links demographic information from the Danish Civil Registration system,[13] chlamydia test data from a purpose-generated dataset of all chlamydia tests performed in public laboratories and healthcare presentations for PID (out-patient, emergency department and in-patient) in the Danish National Patient Register.[14] The dataset has complete ascertainment of chlamydia tests undertaken in public laboratories and diagnosed episodes of PID seen in secondary care (no primary or community care presentations). The Denmark Chlamydia Study contains all women in Denmark (including Greenland) with a positive chlamydia test (1^st^ January 1992 – 2^nd^ November 2011) and four age- and sex-matched controls who were followed up until 2012 (n = 605,475). A cohort of 516,720 women resident in Denmark who entered the dataset between 1995 and 2011 when aged 15–44 years has already been generated from the Denmark Chlamydia study.[5] For this analysis the cohort of 516,720 women was limited to women who were tested for chlamydia (n = 286,223). All women entered the dataset on the date of their first chlamydia test during the study period. Due to the case-control design of the original Denmark Chlamydia Study we were not able to obtain a chlamydia prevalence estimate (*p*_*e*_) from the dataset and instead we use the pooled average estimate from six nationally representative studies of sexually-experienced women aged 18–26 years (2004–2012).[1] We classified women according to the result of their first chlamydia test in the cohort and determined the proportion of chlamydia-negative (*I*_*u*_) and chlamydia-positive women (*I*_*e*_) who had a healthcare presentation with PID by 12-months.

For both datasets we used *p*_*e*_, *I*_*e*_ and *I*_*u*_ to calculate: (1) relative risk (RR) of PID in women who were chlamydia-positive compared to chlamydia-negative at baseline (RR = I_e_/I_u_); (2) attributable risk (AR) of chlamydia on PID (AR = I_e_-I_u_); (3) attributable risk percentage (AR%) of chlamydia on PID (AR% = [(I_e_-I_u_)/I_e_)]*100); and (4) population excess fraction (PEF) of chlamydia on PID (PEF = p_e_(RR-1)**/**[1+p_e_(RR-1)]). We then used the PEF to estimate the number of cases of PID attributable to baseline chlamydia (number of attributable cases = total cases of PID*PEF) and the number of PID cases attributable to baseline chlamydia per 100,000 women tested for chlamydia (attributable case rate = number of attributable cases/size of cohort of tested women*100,000).

### Systematic review

We conducted a systematic search of Embase, Ovid Medline and Pubmed databases in August 2015 to identify published studies of women in the general population that report the risk of PID at 12-months in women tested for chlamydia separately by test result (i.e. the risk of PID in chlamydia-positive and chlamydia-negative women). We searched Pubmed using MESH terms: "chlamydia", or "*Chlamydia trachomatis*", or "chlamydia infections" combined with "pelvic inflammatory disease", or "reproductive health" and relevant study designs: "cohort studies", or "follow-up studies", or "observational study", or "randomized controlled trial", or "epidemiologic studies", or "case-control studies". We used no date restrictions and limited the results to human studies published in English (excluding editorials, abstracts and letters in Ovid) where the full text was available. We adapted this search for Ovid. Studies were eligible if they included: (1) general population of asymptomatic women; (2) tested for chlamydia at baseline; (3) chlamydia-positive and chlamydia-negative women; (4) chlamydia status recorded before PID outcome; (5) incidence of PID at 12-months by chlamydia test result (positive and negative).

**Fig 1:**
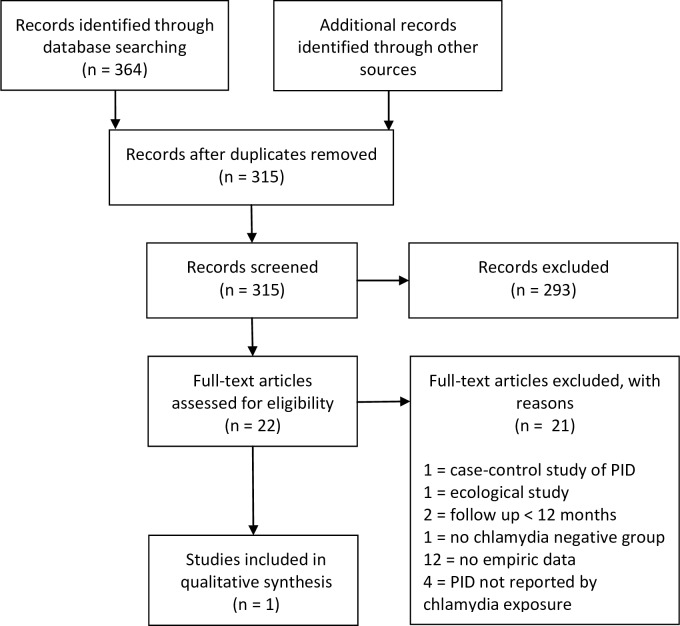
Flowchart of study selection from systematic review.

This identified a single eligible study (Fig 1). The Prevention of Pelvic Infection RCT (POPI-RCT) of Oakeshott *et al*. recruited 2563 women aged 16–27 years from London-based educational settings between 2004–2006.[15] Women were randomised to immediate screening and treatment (screened-arm) or a deferred testing arm (deferred-arm) where chlamydia samples were processed at 12-months. This study was performed before chlamydia screening was rolled-out across England and therefore at the time of the study it was not standard practice to test all asymptomatic young women for chlamydia. At recruitment all women provided a self-taken vaginal swab and at 12-months 2377 women self-reported (or GP in the case of non-respondents) PID or symptoms of PID since recruitment. Separately for the screened- and deferred-arms we extracted *p*_*e*_ (defined as the proportion of women who had a positive result on the sample provided at recruitment), and determined *I*_*e*_ at 12-months. It was not possible to calculate *I*_*u*_ separately for each arm, and a combined figure was determined. We used this information to calculate the RR, AR, AR%, PEF, number of attributable cases, and attributable case rate.

## Results

The three studies are from high income countries (Canada, Denmark, and UK). At the time of the studies, Manitoba and Denmark had widespread chlamydia control interventions including testing for asymptomatic people. The UK (England) had not yet implemented chlamydia screening but widespread testing was undertaken in sexual health clinics (based on risk). The study populations are 2,529 participants (POPI-RCT), 72,883 (Manitoba) and 286,223 (Denmark). The estimated prevalence of chlamydia in women tested for the infection was similar in Manitoba (5.48% (95% CI 5.31–5.65)) and both arms of POPI-RCT (5.93% (4.63–7.23) deferred; 5.42% (4.17–6.68) screened)) and lower for the published estimate used for the Denmark study (4.3% (3.7–5.0)) (Table 2).

**Table 2:** Summary of risk estimates.

	Manitoba, Canada	Denmark Chlamydia Study	UK-based POPI-RCT	
			Deferred screening	Screened
**Prevalence of chlamydia (95% CI)**	5.48% (5.31–5.65)	4.32% (3.65–4.99)	5.93% (4.63–7.23)	5.42% (4.17–6.68)
**Observed rate of PID per 100,000 women by 12-months**	2086.04	890.16	1598.96~	1346.07~
**Incidence of PID in chlamydia positive (95% CI)**	5.27% (4.59–6.00)	1.28% (1.21–1.35)	9.46% (2.79–16.13)	1.59% (0.00–8.53)
**Incidence of PID in chlamydia-negative (95% CI)**	1.90% (1.80–2.00)	0.67% (0.63–0.71)	1.34% (0.91–1.91)*	
**Relative risk (95% CI)**	2.77 (2.41–3.20)	1.92 (1.78–2.08)	7.06 (3.21–15.55)	1.19 (1.64–8.55)
**Attributable risk**	3.37	0.61	8.12	0.25
**Attributable risk %**	63.95%	48.01%	85.84%	15.63%
**Population excess fraction (95% CI)**	8.86% (7.15–10.75)	3.84% (3.26–4.41)	26.44% (11.57–46.32)	0.99% (0.00–29.06)
**Crude number of cases attributed to baseline chlamydia in cohort of tested women (study size)**	134 (72,883)	98 (286,223)	6 (1270)**	0.1 (1259)**
**Number of cases attributed to baseline chlamydia per 100,000 tested women**	184	34	484	13

~Data not available to allocate PID incidence in chlamydia-negative women to the two study arms therefore combined chlamydia-negative data used in calculation

*Data not available to calculate separately

**Assumption that incidence of PID in chlamydia-negative women is 1.34%.

The crude incidence of PID at 12-months in chlamydia-positive women was lowest in Denmark (1326/103,334, 1.28% (95% CI 1.21–1.35)), followed by screened-arm of POPI-RCT (1/63, 1.59% (0.00–8.53), Manitoba (210/3991, 5.27% (4.59–6.00)) and deferred-arm of POPI-RCT (7/74, 9.46% (3.89–18.52)). Chlamydia-negative women had a significantly lower incidence of PID compared to chlamydia-positive women in all studies apart from the screened-arm of POPI-RCT. The RR of PID in a chlamydia-positive woman compared to chlamydia-negative women ranged from 7.06 (95% CI 3.21–15.55) in deferred-arm of POPI-RCT to 1.19 (1.64–8.55) in screened-arm of POPI-RCT (Table 2).

At 12-months, the proportion of cases of PID in treated chlamydia-positive women that could be attributed to a baseline chlamydia infection (AR%) was 15.63% in screened-arm of POPI-RCT, 48.01% in Denmark and 63.95% in Manitoba. The figure for untreated chlamydia positive women was 85.84% in deferred-arm of POPI-RCT. The PEF of chlamydia on PID in the presence of testing and treatment was 8.86% (95% CI 7.15–10.75) in Manitoba, 3.84% (3.26–4.45) in Denmark and 0.99% (0.00–29.06) in screened-arm of POPI-RCT. In the absence of treatment the PEF was 26.44% (95% CI 11.57–46.32) in deferred-arm of POPI-RCT. The PEF of chlamydia on PID in the observational cohorts is between 66.49–85.48% lower than that in the deferred-arm of POPI-RCT.

If all baseline chlamydia infections had been prevented we estimate that per 100,000 women there would have been 13 fewer cases of PID at 12-months in screened-arm of POPI-RCT, 34 fewer in Denmark and 184 in Manitoba. If universal treatment was provided in an untreated setting, we estimate that there would be 484 fewer cases of PID per 100,000 tested women (deferred-arm of POPI-RCT).

## Discussion

### Main findings

In the presence of an established testing and treatment programme (Manitoba and Denmark), less than 10% of diagnosed PID was attributable to baseline treated chlamydia infections. We found that widespread testing and treatment, as it has occurred in practice, may have reduced the PEF of chlamydia on PID by at least 65% compared to the untreated setting. The low PEFs of chlamydia on PID suggest that there are other important causes of PID in all settings and more information is needed about the aetiology of PID to develop effective strategies for improving the reproductive health of women.

### Strengths and limitations

We have compared the PEF from two settings where widespread chlamydia control interventions were present (testing and treatment) to the PEFs from the two arms of a RCT: treated and untreated infection. Limitations in the analysis of the MWRSH cohort and Denmark Chlamydia Study are described in full in the original publications.[5, 6] The main limitation is misclassification bias in chlamydia and PID status. Chlamydia exposure status was assessed at one time-point using imperfect diagnostic tests. Therefore the analysis does not capture changes in exposure status during follow-up (from subsequent diagnosed or undiagnosed infections). The absence of a gold-standard diagnostic test for PID contributes to a diagnostic bias (misclassification bias) towards PID in women with a current or past history of infection compared to women with an equivalent clinical presentation but no documented infection.[16] Under ascertainment of PID diagnoses due to limitations in the available administrative data also results in a misclassification bias in PID status. In this analysis, we assume that all diagnosed chlamydia infections were treated.

The systematic literature search was limited to full-text publications in English language which may have missed potential studies, although these are not known to the authors. The data available for extraction from the POPI-RCT was not complete and we used a single risk of PID in chlamydia-negative women across the two arms. The accuracy of our findings will be affected if this parameter differed. The POPI-RCT was also underpowered to calculate a difference in the incidence of PID across the two arms.[17]

We were not able to calculate the prevalence of chlamydia in tested women in Denmark due to the case-control design of the original study and instead used an estimate of population prevalence from a recent systematic review which may differ from the prevalence in tested women in the general population.[1, 5]

We used RR to describe the difference in the incidence of PID at 12-months in chlamydia-positive and -negative women. This measure does not take into account potential loss to follow-up (e.g. outmigration) but given the short duration of the study (12-months) we assume that loss to follow-up would be low in these large population-based cohorts.

### Comparisons of the included studies

The PEF of chlamydia on PID at 12-months varied across the settings with chlamydia testing and treatment. This variation may be explained by differences in the study populations (e.g. age range of included women; design of healthcare system), available data (e.g. healthcare settings providing data on PID diagnoses) or unmeasured confounders (e.g. incidence of gonorrhoea) rather than an underlying biological difference.

Each study focussed on women of reproductive age, but the age ranges varied (Manitoba 12–24 years at entry; Denmark 15–44 years; POPI-RCT 16–27 years). This is important because PID-diagnosis rates vary with age (highest in 20–24 year olds) and there are age-related patterns in the setting of healthcare presentations (younger women (15–24 years) are more likely to be diagnosed in primary care and older women (25–44 years) are more likely to be diagnosed in the in-patient setting).[18]

The healthcare systems in the three settings differ in their structure, funding and clinical pathways. It is possible that women, with an equivalent clinical presentation, would preferentially attend different healthcare facilities in each setting. Further research is needed to explore the potential impact of this on estimates of PEF. The population-based cohorts contain complete ascertainment of PID diagnoses from the included clinical settings, but these settings varied due to the available data. Ascertainment was likely to be highest in Manitoba (hospital and primary care events), lower in Denmark (hospital care only) and indeterminate for POPI-RCT (self-reported). This could impact the PEF as the likelihood that a presentation with PID occurs in primary care rather than hospital is plausibly related to disease severity (e.g. more severe disease presenting out of hours in Emergency Departments). This in turn could be related to the aetiology of the PID as the risk of hospitalisation is higher for *Neisseria gonorrhoea* (gonorrhoea)-associated PID than chlamydia-associated disease.[19]

There are also differences in the rate of gonorrhoea infection in women in the three settings (Manitoba 105 per 100,000 in 2013[10]; Denmark 4.2 per 100,000 in 2011[11]; UK 25.7 per 100,000 in 2012[12]) and chlamydia-positive women are more likely to be infected with gonorrhoea than chlamydia-negative women.[20] Therefore a higher proportion of the PEF of chlamydia on PID in Manitoba may be due to unknown co-infection with gonorrhoea and/or a higher ascertainment of chlamydia-associated PID compared to the other settings.

There are other factors that it was not possible to quantify from the available data that are likely to contribute to the differences in the observed PEF of chlamydia on PID across the settings. These include the risk profile of women participating with testing (e.g. symptomatic versus asymptomatic) which may be influenced by clinical practice, treatment rate of diagnosed infection, performance of interventions designed to prevent re-infection (e.g. partner notification; health promotion; widespread testing in men), and diagnostic practices of clinicians that affect the likelihood that pelvic pain results in diagnosis of PID.[16, 21]

### Comparison with other studies

A recent modelling study has used multi-parameter evidence synthesis to combine data from multiple sources (including microbiological studies, retrospective case–control studies, RCTs of screening and routine data).[4] Critical estimates used in this modelling study were obtained from the POPI-RCT. The authors estimate that the widely used policy of annual chlamydia testing in young adults can prevent a maximum of 61% of cases of chlamydia-associated PID. We analysed the published data from the POPI-RCT and the retrospective population-based cohorts using a consistent method to improve comparability of the PEF estimates from each setting. Our findings support the predictions from the modelling study. We found that widespread testing and treatment, as it has occurred in practice (Manitoba and Denmark), may have reduced the PEF of chlamydia on PID by at least 65% compared to the untreated setting (POPI-RCT).

### Meanings of the study

The low PEF of diagnosed chlamydia on PID suggests that the aetiology of the majority of diagnosed PID cases is not diagnosed chlamydia. Likely causes include (1) repeat chlamydia infections after the initial test; (2) infection with another causative agent of PID e.g. gonorrhoea or *Mycoplasma genitalium*; (3) diagnostic bias which tends the differential diagnosis of pelvic pain towards PID in women with a past history of chlamydia or chlamydia testing. False negative chlamydia tests at baseline could also explain a small proportion of PID in chlamydia-negative women: if we assume a false positive rate of 2% and a risk of progression to PID for untreated infections of 10% then false negative tests could account for 10% of the observed PID cases in chlamydia-negative women in Manitoba; 30% in Denmark; and 15% in POPI-RCT.

The PEF of chlamydia on PID at 12-months varied between settings with testing and treatment. This suggests that population-specific parameters are required for decision making. The estimate of the PEF from the deferred-arm of the POPI-RCT should be interpreted as a lower estimate of the potential impact of introducing screening into a naïve population as participants were “advised to be screened independently”[15] and testing and treatment outside the study may have occurred. In the settings with widespread control, our study suggests that eradicating all diagnosed infections would result in a small reduction in the incidence of PID (up to 10%). It is possible that expanding interventions may have a further impact on PID by preventing repeat chlamydia infections and infection with other STIs. Mathematical models can be used to explore these assumptions.

Given the low PEF of chlamydia on PID in the settings with widespread control we suggest that a renewed focus on the control of PID (and other post-infectious reproductive complications) should be applied if we wish to improve women’s reproductive health. To achieve this, better information about the contemporary aetiology of PID is needed in order to ensure that prevention efforts tackle PID (and other reproductive complications) rather than chlamydia alone.

### Conclusion

Testing and treating chlamydia infections could reduce the PEF of chlamydia on PID by at least 65%. But in the presence of widespread testing and treatment over 90% of PID cases within 12-months cannot be attributed to a known baseline chlamydia infection. We require more information about the contemporary aetiology of PID in order to develop more effective strategies for improving the reproductive health of women.

## Supporting information

S1 File PRISMA 2009 Statement. (DOC)
